# Epidemiology and antimicrobial susceptibility of invasive *Escherichia coli* infection in neonates from 2012 to 2019 in Xiamen, China

**DOI:** 10.1186/s12879-021-05981-4

**Published:** 2021-03-23

**Authors:** Jidong Lai, Yao Zhu, Lixia Tang, Xinzhu Lin

**Affiliations:** 1 Department of Neonatology, Women and Children’s Hospital, School of Medicine, Xiamen University, No.10 Zhenhai Road, Siming District, Xiamen, 361003 Fujian Province China; 2 Xiamen key laboratory of perinatal-neonatal infection, Xiamen, 361003 Fujian Province China

**Keywords:** *Escherichia coli*, Early-onset infection, Late-onset infection, Clinical characteristics, Drug resistance, Newborn

## Abstract

**Background:**

*Escherichia coli* (*E. coli*) is one of the important causative pathogens of neonatal invasive infection. The epidemiological and clinical profile of invasive *E. coli* infection in Chinese newborns is not well characterized.

**Methods:**

Ninety-four infants with invasive *E. coli* infection were categorized into *E. coli* early onset disease (EOD) group (onset ≤72 h after birth) (*n* = 46) and *E. coli* late onset disease (LOD) group (onset > 72 h) (*n* = 48). We compared and analyzed the clinical characteristics and drug sensitivity profile of early-onset and late-onset *E. coli* invasive infection in neonates.

**Results:**

The incidence of *E. coli*-EOD and *E.coli*-LOD was 0.45/1000 live births (LBs) and 0.47/1000 LBs, respectively. The incidence of gestational diabetes mellitus, perinatal fever, urinary tract infection, chorioamnionitis, and positive *E. coli* culture among mothers in the *E. coli*-EOD group were significantly higher than that in *E. coli*-LOD group. The incidence of premature birth, low-birth-weight, nosocomial infection, and hospitalization time were significantly higher in the *E. coli*-LOD group. The main disease in *E. coli*-EOD group was pneumonia (main clinical manifestation: dyspnea). The main disease in *E. coli*-LOD group was sepsis (main clinical manifestation: fever). The sensitivity rates of *E. coli* strains to ampicillin and piperacillin were low (25.00–28.79%); sensitivity to cephalosporins was also low except ceftazidime (lowest sensitivity rate: 57.14%). Sensitivity to compound preparations containing β-lactamase inhibitors was high, even for extended spectrum β-lactamase-positive strains (nearly 100%).

**Conclusion:**

*E. coli* is an important cause of invasive infection of newborns in Xiamen, China. *E. coli*-EOD was largely attributable to perinatal factors, while *E. coli*-LOD was largely related to nosocomial infection. Compound preparations containing β-lactamase inhibitor or carbapenem antibiotics should be preferred for neonatal invasive infection by *E. coli*.

## Background

According to a meta-analysis, neonatal mortality accounted for 45.1% of deaths in children under the age of 5 years globally in 2015; of these, approximately 21% deaths were caused by neonatal infection including pneumonia, sepsis and purulent meningitis [1]. *Escherichia coli (E. coli)* is a common causative pathogen of neonatal invasive infectious diseases [2, 3]. According to the time of onset of infection, *E. coli* disease is categorized into early-onset *E. coli* disease (*E.coli*-EOD; onset within 3 days after birth) and late-onset *E. coli* disease (*E. coli*-LOD; onset within 3–28 days after birth). Neonatal *E. coli*-EOD is largely caused by prenatal or intrapartum infection (vertical transmission) and typically manifests as pneumonia. *E. coli*-LOD typically presents as sepsis, pneumonia, or meningitis, which is mostly due to environmental or nosocomial bacterial infection [2, 4].

There is considerable variability in the incidence of neonatal invasive *E. coli* infection in different geographical areas and countries. In a study of 30 neonatal intensive care units (NICU) in the United Kingdom (UK), the incidence of early-onset *E. coli* sepsis and late-onset *E. coli* sepsis in the period 2005–2014 was 0.12/1000 live births (LBs) 0.33/1000 LBs, respectively [3]. In an Italian study, the incidence of neonatal late-onset *E. coli* sepsis was found to be 0.35/1000 LBs [5]. In China, *E. coli* is the main causative pathogen of neonatal invasive infection, especially neonatal sepsis [6].

The wide usage of broad-spectrum antibiotics has fueled the emergence of drug-resistant strains of *E. coli*, which poses a great challenge in the treatment of neonatal bacterial infections. Several recent studies have found that *E. coli* is widely resistant to ampicillin and shows different degrees of resistance to the third-generation cephalosporins [2, 7]. However, the drug resistance profile of *E. coli* shows considerable inter-regional variability depending on the local pattern of antibiotic use, which is worthy of further study.

Currently, there is a lack of robust data pertaining to the incidence, clinical characteristics, and drug sensitivity profile of neonatal *E. coli* invasive infection in China. The purpose of this study was to analyze the clinical characteristics and drug sensitivity profile of early-onset and late-onset *E. coli* invasive infection in China. Our findings may provide a scientific basis for more targeted prevention and control measures.

## Methods

### Study population and design

This study was approved by the Ethics Committee of the Women and Children’s Hospital, School of Medicine, Xiamen University (Xiamen, China). We retrospectively reviewed the medical records of 106 infants with invasive *E. coli* infection who were hospitalized at our department between January 2012 and December 2019.

#### Inclusion criteria

Infants with invasive *E. coli* infection including *E. coli* pneumonia, *E. coli* sepsis, *E. coli* meningitis, and *E. coli* abscess.

#### Exclusion criteria

1) Asymptomatic patients with positive *E. coli* culture of the upper airway secretions or body surface secretions; 2) infants with congenital malformations and genetic metabolic diseases; and 3) infants in whom treatment was stopped within 72 h after birth upon request by the family members.

**Fig. 1:**
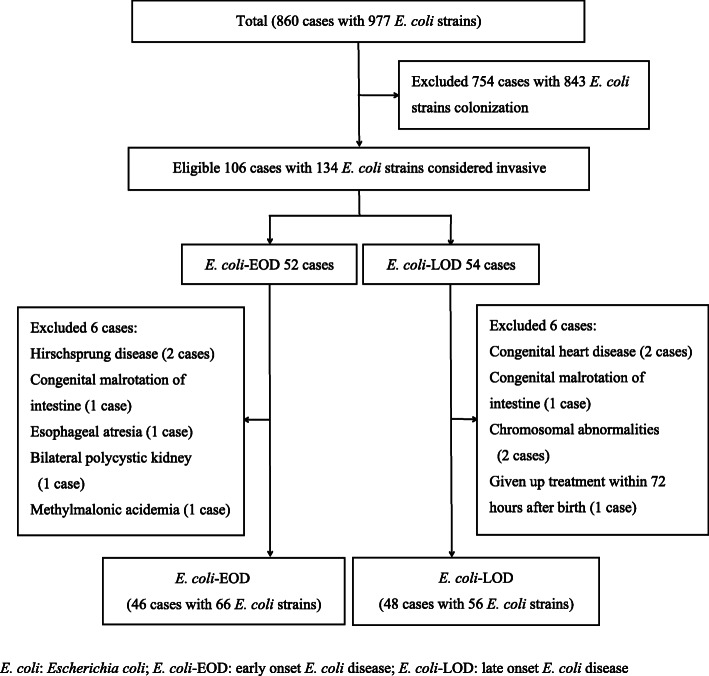
Schematic illustration of the study population and patient-selection criteria. *E. coli*: *Escherichia coli*; *E. coli*-EOD: early onset *E. coli* disease; *E. coli*-LOD: late onset *E. coli* disease

The study subjects were categorized into colonization group and invasive infection group according to the presence or absence of clinical manifestations. The invasive infection group was further categorized into two groups according to the time of onset of the disease. In the *E. coli*-EOD group (*n* = 46), infection occurred within 72 h after birth, while in the *E. coli*-LOD group (*n* = 48), infection occurred within 3–28 days after birth (Fig. 1). Based on the resistance of *E. coli* to extended spectrum β-lactamase (ESBL), the invasive infection group was further categorized into ESBL-positive group and ESBL-negative group.

### Study data set

Demographic and clinical data of subjects were collected using a specially designed questionnaire. The data collected included basic information (sex, gestational age, birth weight, age at admission, age at onset), prenatal conditions [premature rupture of membranes (PROM), meconium-stained amniotic fluid (MSAF), intrauterine infection, prenatal hormone use, gestational diabetes mellitus (GDM)], spectrum of disease, complications, drug sensitivity results, treatment, and outcomes.

### *E.coli* culture and antimicrobial susceptibility testing

Prior to administering antibiotics, samples of tracheal secretion, peripheral blood, cerebrospinal fluid, pus, or urine were cultured in Columbia blood agar plate (Beiruite Biotechnology Co. Ltd., Zhengzhou, China) for bacterial identification and determination of the *E. coli* strains using the American PHOENIX100 bacterial identification system (Becton and Dickinson Company, Franklin Lake, NJ, USA). After identification, *E. coli* strains were isolated and drug sensitivity test was carried out by disk diffusion method. All disks were purchased from the Mast Co. UK. Antibiotic sensitivity testing was carried out as recommended by the Clinical and Laboratory Standards Institute (CLSI), United States. *E. coli* ATCC 25922 was used for quality control of the test provided by the clinical laboratory center of the Chinese Ministry of health. If the diameter of the inhibition zone around the cefotaxime/clavulanic acid and ceftazidime/clavulanic acid disks was at least 5 mm greater compared to disks without clavulanic acid, the strain was considered as ESBL-producer.

### Study definitions/diagnostic criteria

#### *E. coli* invasive infection

Positive *E. coli* culture from tracheal secretions (collected by endotracheal intubation within 30 min after admission and prior to antibiotic use), blood, pus, or urine collected from infants aged 1–28 days with symptoms and diagnosis of pneumonia (shortness of breath, exudative shadows in X-ray images, positive *E. coli* culture from tracheal secretions), sepsis, meningitis, urinary infection (fever, abnormal urine routine, positive *E. coli* culture from urine) or skin abscess (fever, swelling, and tenderness, positive *E. coli* culture from pus). *E. coli* invasive infection was diagnosed according to the Practical Neonatology (5th ed.) and the Chinese neonatal sepsis guidelines [8, 9].

#### Complications of invasive *E. coli* infection

Based on the diagnosis of invasive *E. coli* infection, occurrence of the following complications was defined as severe *E. coli* infection: 1) Acute respiratory distress syndrome (ARDS) according to the Montreux Standard (2017 Edition) [10]; 2) Pulmonary hemorrhage according to Practical Neonatology (5th ed.) [8]; 3) Septic shock in line with the guidelines for septic shock in children [11]; 4) Persistent pulmonary hypertension of the newborn (PPHN) consistent with the diagnostic criteria of neonatal pulmonary hypertension [12]; 5) Necrotizing enterocolitis (NEC) according to Practical Neonatology (5th ed.) [8].

### Statistical analyses

SPSS Statistics for Windows, Version 25.0 (IBM Corp., Armonk, NY, USA) was used for statistical analysis. Distribution of the data was assessed for normality using the Kolmogorov-Smirnov test. Normally distributed continuous variables are presented as mean and standard deviation, and between-group differences assessed using the Student’s *t* test. Categorical variables are presented as frequency and percentage and between-group differences assessed using the Chi-squared or Fisher’s Exact test. Non-normally distributed continuous variables are presented as median and interquartile range [M (P25-P75)], and between-group differences assessed using the Mann Whitney *U* test. *P* values < 0.05 were considered indicative of statistical significance.

## Results

### Frequency of *E. coli* colonization and invasive infection in newborns

During the study reference period, a total of 115,538 live births (LBs) occurred in our hospital, of which 36,310 newborns were hospitalized in our department. Among these, 977 *E. coli* strains were isolated from 860 newborns. A total of 754 cases with 843 strains had no clinical manifestations and were considered as cases of *E. coli* colonization, while 106 cases with 134 strains were considered as cases of *E. coli* infection. Of these, 12 cases were excluded based on our study-selection criteria. Thus, 94 infants with 122 *E. coli* strains isolated from various specimens were included in this study; of these, 46 were categorized as *E. coli*-EOD cases and 48 as *E. coli*-EOD cases. The detection rate of *E. coli* from newborns at our hospital was 7.44/1000 LBs (860/115538) and 23.68/1000 neonatal admissions (NAs) (860/36310); the incidence of neonatal *E. coli* infection was 0.92/1000 LBs (106/115538) and 2.92/1000 NAs (106/36310). The incidence of neonatal *E. coli*-EOD was 0.45/1000 LBs (52/115538) and 1.43/1000 NAs (52/36310), while the incidence of neonatal *E. coli*-LOD was 0.47/1000 LBs (54/115538) and 1.49/1000 NAs (54/36310). The incidence of *E. coli* early-onset sepsis was 0.16/1000 LBs (19/115538) and 0.52/1000 NAs (19/36310), while the incidence of *E. coli* late-onset sepsis was 0.25/1000 LBs (29/115538) and 0.80/1000 NAs (29/36310).

**Fig. 2:**
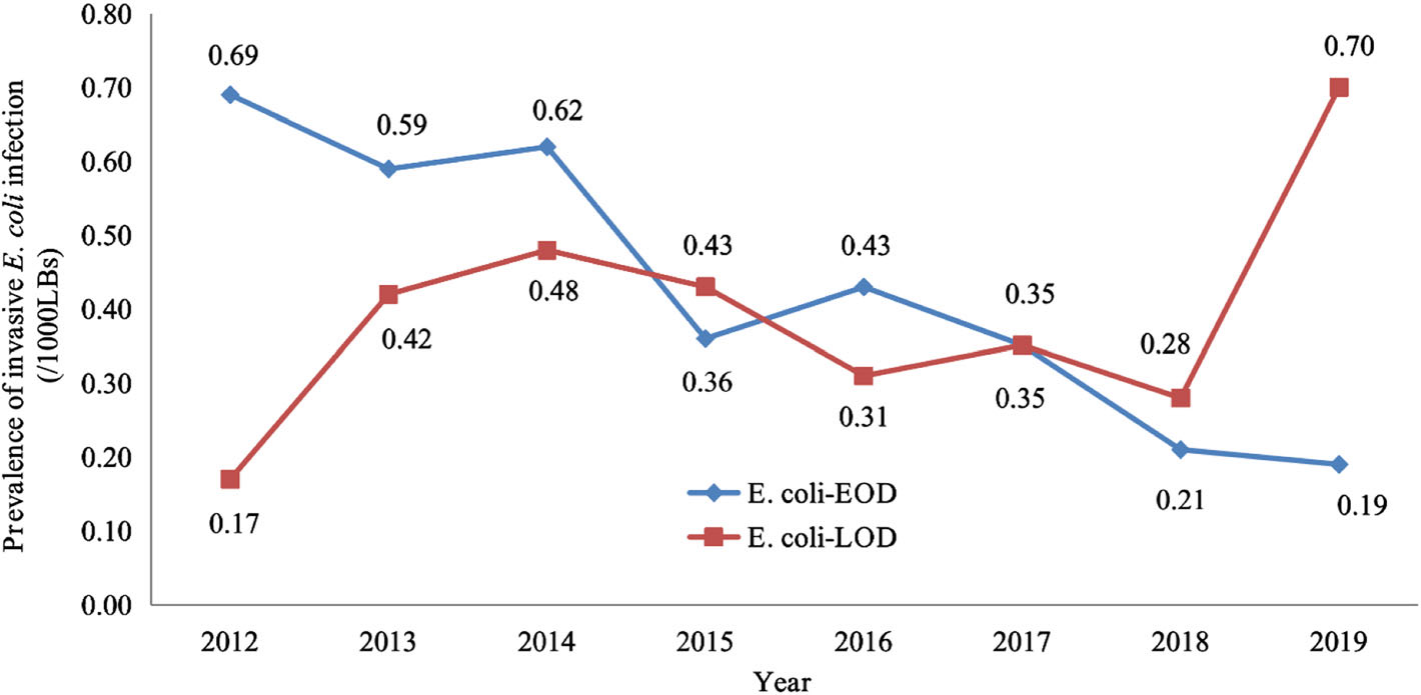
The incidence of *E. coli*-EOD and *E. coli*-LOD over 8 years (2012–2019)

The incidence of *E. coli*-EOD and *E. coli*-LOD in newborns from 2012 to 2019 is shown in Fig. 2. The incidence of *E. coli*-EOD showed a downward trend, gradually decreasing from 0.69/1000 LBs in 2012 to 0.19/1000 LBs in 2019. The lowest incidence of *E. coli*-LOD was in 2012 (0.17/1000 LBs), while the highest incidence was in 2019 (0.70/1000 LBs). The annual incidence in the intervening years showed no major changes and fluctuated within the range of 0.28–0.48/1000 LBs.

### Comparison of clinical features between *E. coli*-EOD group and *E. coli*-LOD group

The average age at onset was 0.50 (0.20, 0.88) h in the *E. coli-*EOD group and 13.52 (7.75, 18.50) d in the *E. coli*-LOD group. In the *E. coli-*EOD group, all cases had onset within 24 h after birth; 39 cases (84.78%) had onset within 2 h after birth. In terms of maternal factors, there were significant differences between the two groups with respect to the incidence of prenatal hormone usage, gestational diabetes mellitus (GDM), intrapartum temperature ≥ 37.5 °C, gestational vaginitis, gestational bacteriuria, chorioamnionitis, and positive *E. coli* culture in placental swab or cervical secretions. In terms of newborns, there were significant differences with respect to gestational age, birth weight, length of hospital stay, incidence of preterm birth, low-birth-weight, meconium-stained amniotic fluid (MSAF), breastfeeding, mechanical ventilation, dyspnea, fever, and nosocomial infection. There were no significant between-group differences with respect to the other clinical features (Table 1).

**Table 1:** Comparison of clinical features between *E. coli*-EOD group and *E. coli*-LOD group

Characteristics	*E. coli*-EOD (*n* = 46)	*E. coli*-LOD (*n* = 48)	χ2/*t*/*z* value	*P*
***Maternal***				
Age, n (%)				
< 20 years	2 (4.35)	1 (2.08)	0.001	0.970
≥ 35 years	4 (8.70)	8 (16.67)	1.34	0.247
Regular antenatal screening, n (%)	38 (82.61)	41 (85.42)	0.138	0.710
Cesarean section, n (%)	20 (43.48)	14 (29.17)	2.081	0.155
Prenatal hormone usage, n (%)	4 (8.70)	14 (29.17)	5.362	0.020
PROM, n (%)	8 (17.39)	7 (14.58)	0.138	0.710
GDM, n (%)	17 (36.96)	4 (8.33)	11.091	0.001
Intrapartum temperature ≥ 37.5 °C, n (%)	24 (52.17)	1 (2.08)	30.189	0.000
Gestational vaginitis, n (%)	10 (21.74)	2 (4.17)	6.513	0.011
Gestational bacteriuria, n (%)	7 (15.22)	0 (0.00)	5.839	0.016
Chorioamnionitis, n (%)	28 (60.87)	9 (18.75)	17.459	0.000
Positive *E. coli* culture in placental swab or cervical secretions, n (%)	21 (45.65)	1 (2.08)	24.873	0.000
***Neonatal***				
Gender (case, male / female)	30/16	31/17	0.021	0.880
Gestational age, median (IQR), weeks	39.8 (39,40.7)	37.1 (32.73,39.28)	4.253	0.000
Preterm, n (%)	4 (8.70)	20 (41.67)	13.433	0.000
Birth weight, median (IQR), grams	3400 (2983,3663)	2875 (1765,3375)	3.138	0.002
Low birth weight, n (%)	4 (8.70)	17 (35.42)	9.667	0.002
Small for gestational age, n (%)	0 (0.00)	2 (4.17)	–	0.495^a^
Asphyxia, n (%)	3 (6.52)	6 (12.5)	0.402	0.526
MSAF, n (%)	26 (56.52)	1 (2.08)	34.002	0.000
Breast-feeding, n (%)	40 (86.96)	32 (66.67)	5.394	0.022
Mechanical ventilation, n (%)	25 (54.35)	7 (14.58)	16.542	0.000
Duration of mechanical ventilation, median (IQR), days	3.0 (1.5,4.5)	5.0 (3.0,10.0)	0.754	0.456
Shortness of breath, n (%)	38 (82.61)	16 (33.33)	23.330	0.000
Fever, n (%)	2 (4.35)	26 (54.17)	27.880	0.000
Convulsions, n (%)	2 (4.35)	2 (4.17)	0.000	1.000
Poor response, n (%)	3 (6.52)	3 (6.25)	0.000	1.000
Jaundice, n (%)	0 (0.00)	1 (2.08)	–	1.000^a^
Nosocomial infection, n (%)	0 (0.00)	18 (37.50)	21.340	0.000
Duration of UVC, mean ± SD, days	4.00 ± 1.79	6.11 ± 3.14	1.483	0.162
Duration of PICC, median (IQR), days	30.00 (10.50,67.00)	28.00 (17.00,52.50)	0.153	0.881
Length of hospitalization, median (IQR), days	13.5 (8,16)	16 (14,30)	2.794	0.005
Mortality, n (%)	3 (6.52)	2 (5.32)	0.002	0.961

*PROM* premature rupture of membranes, *GDM* gestational diabetes mellitus, *MSAF* meconium-stained amniotic fluid, *UVC* umbilical vein catheterization, *PICC* peripherally inserted central catheter

^a^Fisher’s exact test

### Comparison of disease spectrum and complications between *E. coli*-EOD group and *E. coli*-LOD group

The distribution of the disease spectrum of *E. coli* invasive infection was as follows: 1) *E. coli*-EOD group: 30 cases of pneumonia, 10 cases of sepsis, 3 cases of pneumonia+sepsis, 1 case of pneumonia+sepsis+purulent meningitis, 5 cases of sepsis+purulent meningitis; 2) *E. coli*-LOD group: 14 cases of pneumonia, 18 cases of sepsis, 4 cases of urinary tract infection, 3 cases of skin abscess, 2 cases of pneumonia+sepsis, 2 cases of pneumonia+sepsis+purulent meningitis, 5 cases of sepsis+purulent meningitis, 1 case of sepsis+urinary tract infection, and 1 case of sepsis+skin abscess. There was a significant between-group difference with respect to the incidence of pneumonia and sepsis. There were significant between-group differences with respect to the incidence of ARDS, pulmonary hemorrhage, septic shock, and PPHN (Table 2).

**Table 2:** Disease spectrum and complications in the *E. coli*-EOD and *E. coli*-LOD groups

Disease	*E. coli*-EOD (*n =* 46)	*E. coli*-LOD (*n =* 48)	χ2 values	*P*
Pneumonia, n (%)	34 (73.91)	18 (37.50)	12.600	0.000
Sepsis, n (%)	19 (41.30)	29 (60.42)	5.112	0.021
Purulent meningitis, n (%)	6 (13.04)	7 (14.58)	0.050	0.831
Urinary tract infection, n (%)	0 (0.00)	5 (10.42)	–	0.060^a^
Skin abscess, n (%)	0 (0.00)	4 (8.33)	–	0.121^a^
ARDS,n (%)	15 (32.61)	2 (4.17)	12.831	0.000
Pulmonary hemorrhage, n (%)	10 (21.74)	1 (2.08)	8.782	0.003
Septic shock, n (%)	11 (23.91)	2 (4.17)	7.690	0.006
PPHN, n (%)	5 (10.87)	0 (0)	–	0.030^a^
NEC, n (%)	1 (2.17)	3 (6.25)	0.219	0.640^a^

*ARDS* acute respiratory distress syndrome, *PPHN* persistent pulmonary hypertension of the newborn, *NEC* necrotizing enterocolitis

^a^Fisher’s exact test

### Treatment and outcomes of *E. coli-*EOD group and *E. coli-*LOD group

In this study, 61 cases (64.89%) were treated with piperacillin tazobactam, 24 cases (25.53%) with cefoperazone sulbactam, and 9 cases (9.57%) with meropenem.

In the *E. coli*-EOD group, the cure rate was 93.48% (43/46) and the mortality rate was 6.52% (3/46). All fatal cases were diagnosed as pneumonia+sepsis and had complications of ARDS, pulmonary hemorrhage, PPHN, and/or septic shock; in all cases, death occurred within 12 h after onset. In the *E. coli*-LOD group, the cure rate was 95.83% (46/48) and the mortality rate was 4.16% (2/48). All fatal cases were diagnosed as sepsis and had complications of septic shock; in all cases, death occurred within 24 h after onset. The overall mortality rate was 5.32% (5/94). There was no significant between-group difference with respect to mortality (*P* = 0.961).

All 13 cases of purulent meningitis were cured (cure rate: 100%). The findings of plain and contrast-enhanced MRI of brain were consistent with purulent meningitis; these included 2 cases of ependymitis and 1 case of subarachnoid hemorrhage which resolved one month after discharge.

### Antibiotic sensitivity profile in the colonization group and invasive infection group

During the study reference period, 843 strains of *E. coli* cultured from all hospitalized infants in our hospital were considered cases of colonization; these included 592 strains cultured from sputum, 28 strains from endotracheal secretions, 172 strains from gastric secretion, 4 strains from blood, 5 strains from urine, 19 strains from eye secretion, and 23 strains from umbilical secretion. A total of 122 *E. coli* strains were cultured from patients with a diagnosis of invasive infection; these included 66 strains in the EOD group (7 strains from gastric secretion, 34 strains from endotracheal secretion, 19 strains from blood, and 6 strains from the cerebrospinal fluid) and 56 strains in the LOD group (10 strains from endotracheal secretion, 29 strains from blood, 5 strains from urine, 7 strains from CSF, 4 strains from pus, and 1 strain from peripherally inserted central catheter). The positivity rates of ESBL in *E. coli-*EOD group and *E. coli-*LOD group were 25.76% (17/66) and 32.14% (18/56), respectively, with no significant between-group difference (χ^2^ = 0.604, *P* = 0.437). It should be noted that ESBL of *E. coli* was negative in all the 5 patients who died.

Sensitivity to cephalosporins (including cefazolin, ceftazidime, cefotaxime, and cefepime) and aztreonam was lower in the colonization group compared with the invasive infection group. The sensitivity rates of cefepime and amoxicillin/clavulanic acid in the EOD group were higher than those in the colonization group and LOD group (*P* = 0.023 and 0.021, respectively); there was no significant difference with respect to sensitivity to other antibiotics. None of the strains in either group were resistant to amikacin, imipenem, or meropenem.

Compared with the ESBL-negative group, the antibiotic sensitivity rate in the ESBL-positive group was lower, except for compound preparation of β-lactamase inhibitors (ampicillin/sulbactam, cefoperazone/sulbactam and piperacillin/tazobactam). None of the strains in the ESBL-positive group were resistant to amikacin, imipenem, or meropenem (Tables 3 and 4).

**Table 3:** Antibiotic sensitivity profile of *Escherichia coli* in the colonization group and invasive infection group [% (*n*/N)]

Antibiotic		Colonization group (*n* = 843)	Invasive infection group		χ^2^ values	*P*
			*E. coli*-EOD (*n* = 66)	*E. coli*-LOD (*n* = 56)		
β-lactams	Ampicillin	23.49 (198/843)	28.79 (19/66)	25.00 (14/56)	0.981	0.612
	Piperacillin	23.84 (201/843)	28.79 (19/66)	26.79 (15/56)	1.000	0.610
	Cefazolin	46.26 (390/843)	66.67 (44/66)	57.14 (32/56)	12.073	0.002
	Ceftazidime	79.48 (670/843)	95.45 (63/66)	91.07 (51/56)	14.024	0.001
	Cefotaxime	58.24 (491/843)	86.36 (57/66)	73.21 (41/56)	24.063	0.000
	Cefepime	61.92 (522/843)	87.88 (58/66)	71.43 (40/56)	19.290	0.000
Compound preparations of β-lactam inhibitors	Amoxicillin/clavulanic acid	70.23 (592/843)	89.39 (59/66)	73.21 (41/56)	11.148	0.004
	Ampicillin/sulbactam	47.57 (401/843)	50.00 (33/66)	55.36 (31/56)	1.370	0.501
	Cefoperazone /sulbactam	100.00 (843/843)	100.00 (66/66)	98.21 (55/56)	–	–
	Piperacillin/tazobactam	95.14 (802/843)	100.00 (66/66)	94.64 (53/56)	3.412	0.184
Carbapenems	Imipenem	100.00 (843/843)	100.00 (66/66)	100.00 (56/56)	–	–
	Meropenem	100.00 (843/843)	100.00 (66/66)	100.00 (56/56)	–	–
Quinolones	Levofloxacin	74.61 (629/843)	68.18 (45/66)	67.86 (38/56)	2.392	0.303
	Ciprofloxacin	69.75 (588/843)	65.15 (43/66)	62.50 (35/56)	1.791	0.412
Aminoglycosides	Gentamicin	72.84 (614/843)	78.79 (52/66)	83.93 (47/56)	4.230	0.124
	Amikacin	100.00 (843/843)	100.00 (66/66)	100.00 (56/56)	–	–
Sulfonamides	Compound sulfamethoxazole	52.31 (441/843)	51.52 (34/66)	55.36 (31/56)	0.217	0.892
Other antibiotics	Aztreonam	69.51 (586/843)	93.94 (62/66)	83.93 (47/56)	28.223	0.000

**Table 4:** Antibiotic sensitivity of infants with ESBL-positive and ESBL-negative *Escherichia coli* invasive infection [% (*n*/N)]

Antibiotic		ESBL positive group (*n* = 35)	ESBL negative group (*n* = 87)	χ^2^ values	*P*
β-lactams	Ampicillin	2.86 (1/35)	36.78 (32/87)	14.557	0.000
	Piperacillin	8.57 (3/35)	35.63 (31/87)	9.092	0.003
	Cefazolin	11.43 (4/35)	82.76 (72/87)	54.066	0.000
	Ceftazidime	80.00 (28/35)	98.85 (86/87)	11.561	0.001
	Cefotaxime	37.14 (13/35)	97.70 (85/87)	57.924	0.000
	Cefepime	34.29 (12/35)	98.85 (86/87)	65.842	0.000
Compound preparations of β-lactam inhibitors	Amoxicillin/clavulanic acid	57.14 (20/35)	91.95 (80/87)	20.463	0.000
	Ampicillin/sulbactam	40.00 (14/35)	57.47 (50/87)	3.055	0.080
	Cefoperazone/sulbactam	97.14 (34/35)	100.00 (87/87)	–	0.287^a^
	Piperacillin/tazobactam	94.29 (33/35)	100.00 (87/87)	–	0.081^a^
Carbapenems	Imipenem	100.00 (35/35)	100.00 (87/87)	–	–
	Meropenem	100.00 (35/35)	100.00 (87/87)	–	–
Quinolones	Levofloxacin	54.29 (19/35)	73.56 (64/87)	4.265	0.039
	Ciprofloxacin	48.57 (17/35)	70.11 (61/87)	5.024	0.025
Aminoglycosides	Gentamicin	60.00 (21/35)	89.66 (78/87)	14.348	0.000
	Amikacin	100.00 (35/35)	100.00 (87/87)	–	–
Sulfonamides	Compound sulfamethoxazole	28.57 (10/35)	63.22 (55/87)	12.036	0.001
Other antibiotics	Aztreonam	65.71 (23/35)	98.85 (86/87)	25.411	0.000

*ESBL* extended spectrum β-lactamase

^a^Fisher’s exact test

## Discussion

In this study, the incidence of *E. coli* invasive infection was 0.92/1000 LBs and 2.92/1000 NAs. The incidence of *E. coli-*EOD was 0.45/1000 LBs and 1.43/1000 NAs, while the incidence of *E. coli-*LOD was 0.47/1000 LBs and 1.49/1000 NAs. The incidence of *E. coli* early-onset sepsis was 0.16/1000 LBs and 0.52/1000 NAs, while the incidence of *E. coli* late-onset sepsis was 0.25/1000 LBs and 0.80/1000 NAs. There is some variability in the incidence rates of neonatal invasive *E. coli* infection in different countries or regions. In a single-center study conducted in Spain spanning the past 20 years, the incidence of neonatal *E. coli* early-onset sepsis was 0.69/1000 LBs [13]. In a study of 21 neonatal care centers in Israel from 2010 to 2015, the incidence of *E. coli* early-onset infection was 0.2/1000 LBs [14]. In a study conducted at a tertiary hospital in India, the rate of neonatal sepsis with blood culture positivity was 6.2%, of which *E. coli* accounted for 14% [15]. In a study conducted across 25 tertiary hospitals in China from 2015 to 2018, the incidence of neonatal early-onset sepsis caused by *E. coli* in preterm infants younger than 34 weeks of gestation was 2.36/1000 LBs [6]. In recent years, *E. coli* has replaced group B streptococcus as the most common causative pathogen of neonatal purulent meningitis in Taiwan [16]. Our study also found a downward trend in the incidence of neonatal *E. coli-*EOD over successive years, and the condition often occurred in term infants (up to 91%). However, the incidence of neonatal *E. coli-*LOD showed no significant change over the years except for a peak in 2019. This phenomenon may be attributable to several factors: 1) The rate of antibiotic usage in mothers in our hospital was high. For example, from 2010 to 2015, the antibiotic use rate of mothers with newborns suffering from early-onset sepsis increased to 38% [17]. Prophylactic use of cefoxitin sodium was more commonly used in cases of threatened preterm labor, which largely eliminated *E. coli* colonization and reduced the vertical transmission from mothers to newborns. 2) Neonatal *E. coli-*LOD is often caused by nosocomial infection, which usually occurs in premature infants or low-birth-weight infants. The peak in the incidence of *E. coli-*LOD in 2019 was related to more premature births and infants with critical diseases, as well as the increase in invasive operations.

Our analysis revealed that the occurrence of *E. coli-*EOD was closely related to perinatal infection, such as fever, gestational vaginitis, gestational bacteriuria, MSAF, and chorioamnionitis. *E. coli* was cultured from 45.65% of mothers and 60.87% of mothers developed chorioamnionitis; this indicated that *E. coli* colonization or infection in the urogenital system of pregnant women can cause intrauterine infection [18]. In our study, the incidence of GDM in mothers with *E. coli-*EOD newborns was significantly higher than that in mothers with *E. coli-*LOD. GDM may affect the development of fetal thymus and immune system, which can induce excessive inflammatory reaction in newborn through innate immune response mediated by TLR5 or TLR1/2 [19, 20]. We found that the incidence of neonatal *E. coli-*LOD was associated with prematurity, low-birth-weight infants, and low breastfeeding rate; in addition, most of these cases suffered from nosocomial infection, which was consistent with a previous report [3]. The immature immune status of premature or low-birth-weight infants, the typically long hospital stay, and lack of breastfeeding render these infants more vulnerable to *E. coli* infection.

In our study, *E. coli-*EOD often manifested as pneumonia with the main clinical manifestations of tachypnea; these infants were prone to complications such as ARDS, septic shock, pulmonary hemorrhage, and PPHN. However, *E. coli-*LOD typically manifested as sepsis, with fever as the main symptom, which was consistent with the disease spectrum and complications caused by intrauterine infection and nosocomial infection. These findings are consistent with those of previous studies [2, 21]. There was no significant difference in the incidence of purulent meningitis between the *E. coli-*EOD group and *E. coli-*LOD group, which was inconsistent with other reports [22, 23]. Previous studies have shown that *E. coli* containing polysaccharide capsule K1 antigen has anti-phagocytotic, anti-antibody and complement functions, leading to strong virulence and pathogenicity; thus, it is more likely to cause purulent meningitis [22]. *E. coli* carrying the *einv* virulence gene easily cause bloodstream-related infection, while *CNF1* and *CNF2* virulence genes may be closely related to the severity of lung damage and multiple organ damage [24]. Therefore, the difference of disease spectrum and complications in *E. coli-*EOD and *E. coli-*LOD may be caused by different virulence genes carried by *E. coli* [25].

Characterization of the drug sensitivity profile of local *E. coli* strains is a key imperative since antibiotic therapy is the mainstay of treatment for *E. coli* invasive infection. In our study, the sensitivity rate of *E. coli* to ampicillin and piperacillin was as low as 23.49–28.79%, while the sensitivity rates to cephalosporins other than ceftazidime were also low, especially in ESBL-positive *E. coli*. *E. coli* strains were found to be highly sensitive to compound preparations including β-lactamase inhibitor; in addition, no carbapenem- or amikacin-resistant strains were found in our study population. The above results were consistent with most previous reports [26, 27]. The sensitivity rates for quinolones and sulfonamides were approximately 70 and 50%, respectively, which was significantly lower than that in a previous report [28]. The difference of antibiotic sensitivity to *E. coli* strains may be related to regional differences, and differences with respect to detection methods and antibiotic use habits. Interestingly, our study showed that the antimicrobial sensitivity of *E. coli* strains causing colonization was generally lower than that of invasive infection strains, including a significant difference with respect to the sensitivity to cephalosporins. This was because most bacterial colonization represented the surviving bacteria after overuse of antibiotics, while the invasive infection strains were mostly mediated by virulence factors. Therefore, antibiotics containing β-lactamase inhibitors such as piperacillin/tazobactam should be the first-choice treatment for *E. coli* invasive infection in Xiamen, China. Carbapenems such as meropenem should be considered only when the therapeutic effect is poor.

The widespread use of broad-spectrum antibiotics, especially the abuse of third-generation cephalosporins, is the main reason for the increase of ESBL producing strains. The mechanism of drug resistance of ESBL-positive *E. coli* strains is drug-induced target gene mutation; in addition, plasmid-mediated transmission among different strains leads to wide spread of drug-resistant strains [29]. In our study, the detection rates of ESBL in *E. coli-*EOD and *E. coli-*LOD groups were 25.76 and 32.14%, respectively. These figures were significantly lower than those reported in other regions of China (45.4%) and in studies conducted overseas (53%) [29, 30]. This is likely attributable to less frequent use of cephalosporins in the neonatal department of our hospital, more intensive monitoring of bacterial resistance, and to the regional differences. In our study, the ESBL-positivity rate in *E. coli-*LOD was higher, and most of the cases were premature infants or low-birth-weight infants with nosocomial infection. The risk factors for occurrence of multi-drug resistant strains were premature delivery, low birth weight, invasive operation, and prolonged hospital stay. Therefore, premature or low-birth-weight infants with long hospitalization stay should be considered at high risk of ESBL-positive *E. coli* invasive infection.

Some limitations of our study should be acknowledged. This was a single-center study, and we did not investigate the serotype and distribution of virulence genes of *E. coli*. Larger multi-center studies involving in-depth exploration at the level of molecular genotype are required for a more comprehensive analysis of invasive *E. coli* infection.

## Conclusion

Our findings suggest that *E. coli* is an important causative pathogen of neonatal invasive infection in Xiamen, China. The clinical manifestations and the disease spectrum of neonatal *E. coli-*EOD were different from those of *E. coli-*LOD. The occurrence of *E. coli-*EOD was mostly related to perinatal factors, while *E. coli-*LOD was mostly related to acquired infection, especially nosocomial infection. Compound preparations containing β-lactamase inhibitor or carbapenems should be preferred for the treatment of neonatal *E. coli* invasive infection, especially for the ESBL-positive strains. This study provided epidemiological data pertaining to *E. coli* for further in-depth study of *s*erotypes and genotypes, drug resistance genes, and virulence genes.

## Data Availability

The datasets generated and analyzed during the current study are available from the corresponding author on reasonable request.

## Abbreviations

*E. coli*: *Escherichia coli*
EOD: Early onset disease
LOD: Late onset disease
NICU: Neonatal intensive care unit
LBs: Live births
NAs: Neonatal admissions
ESBL: Extended spectrum β-lactamase
PROM: Premature rupture of membranes
MSAF: Meconium-stained amniotic fluid
GDM: Gestational diabetes mellitus
CLSI: Clinical and Laboratory Standards Institute
ARDS: Acute respiratory distress syndrome
PPHN: Persistent pulmonary hypertension of the newborn
NEC: Necrotizing enterocolitis
SD: Standard deviation
IQR: Interquartile range
UVC: Umbilical vein catheterization
PICC: Peripherally inserted central catheter
